# Inferring Boolean network states from partial information

**DOI:** 10.1186/1687-4153-2013-11

**Published:** 2013-09-05

**Authors:** Guy Karlebach

**Affiliations:** 1 German Cancer Research Institute (DKFZ), Im Neuenheimer Feld 280, Heidelberg 69121, Germany

**Keywords:** Boolean network, Inference, Conditional entropy, Gradient descent

## Abstract

Networks of molecular interactions regulate key processes in living cells. Therefore, understanding their functionality is a high priority in advancing biological knowledge. Boolean networks are often used to describe cellular networks mathematically and are fitted to experimental datasets. The fitting often results in ambiguities since the interpretation of the measurements is not straightforward and since the data contain noise. In order to facilitate a more reliable mapping between datasets and Boolean networks, we develop an algorithm that infers network trajectories from a dataset distorted by noise. We analyze our algorithm theoretically and demonstrate its accuracy using simulation and microarray expression data.

## Introduction

**Figure 1:**
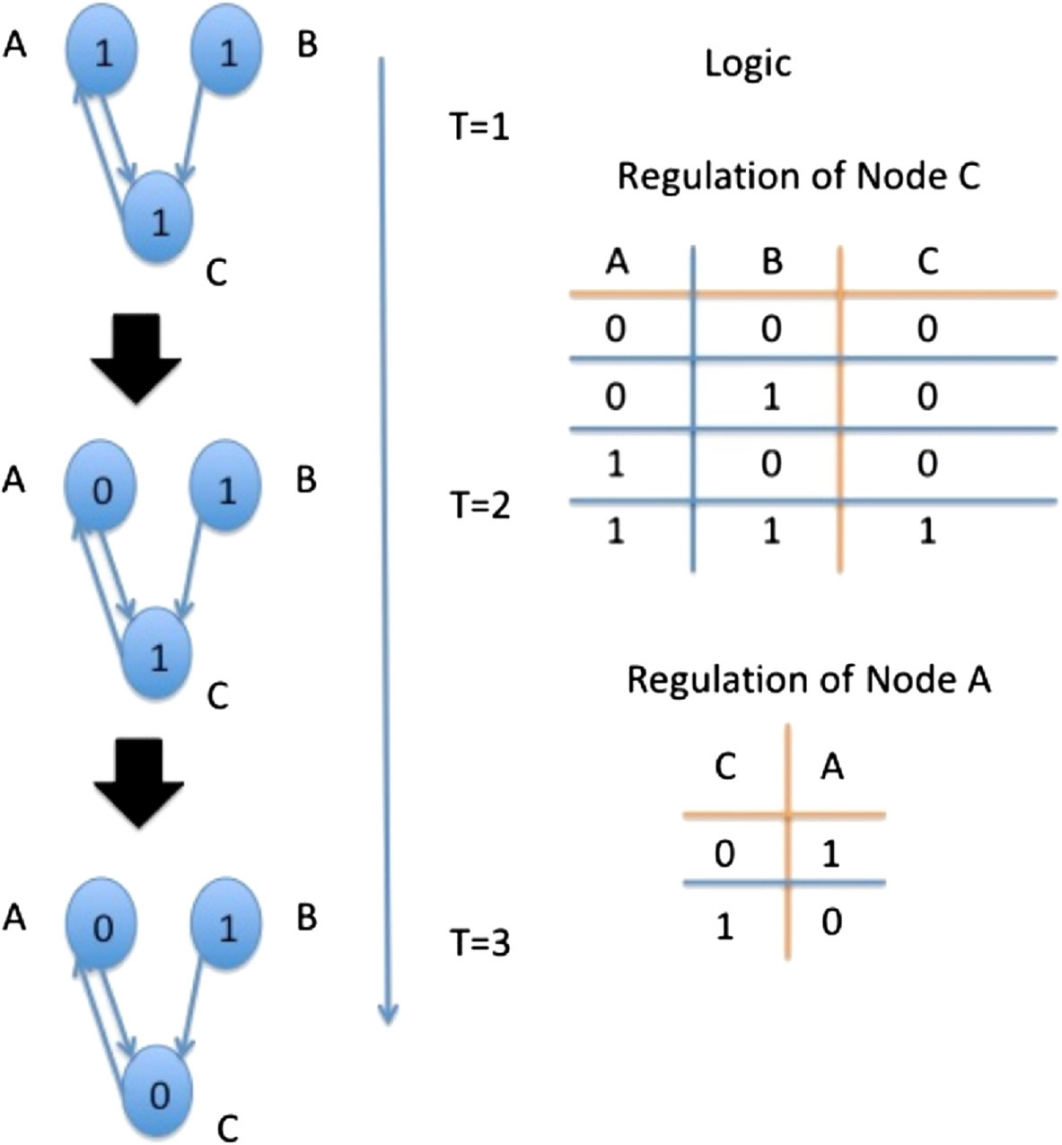
**A simple Boolean network.** The network has three nodes, denoted as **A**, **B**, and **C**. Nodes **A** and **B** can change the Boolean value of node **C**, according to the rules given at the right side of the figure. Similarly, node **C** can change the value of node **A**. At the left side of the figure, a trajectory of length 3 is illustrated. In the initial state (*T* = 1), all the nodes receive a Boolean 1. According to the logical rule by which **C** changes the value of **A**, the value of **A** changes to 0 at time *T* = 2. At time point *T* = 3, the node **C** changes its value, since the input to the logical rule that determines its value has changed.

Boolean networks were introduced by Kauffman [1] several decades ago as a model for gene regulatory networks. In this model, every gene corresponds to a node in the network. Every node is assigned an initial Boolean value, which is its value at time 0. A Boolean value of 1 means that the gene is active; in other words, its product is present in the cell and can perform its designated role. A Boolean value of 0 means exactly the opposite - a gene is not active and its product is absent from the cell. Since the activity or inactivity of genes affects the activity or inactivity of other genes, the Boolean value of a node at time point *T* + 1 is determined by the Boolean values of other nodes at time *T*. More specifically, the Boolean value of a node is determined by a time-invariant Boolean function that takes as input the Boolean values of a set of network nodes at the preceding time point. The set of nodes that constitute the input to the Boolean function is called its regulators, and the output node is referred to as target. The vector of the Boolean values of all the network nodes is called the network state. A sequence of states that evolves from some initial state according to the Boolean functions is called a trajectory. The trajectories of network states can be complex, displaying chaos or order depending on the network structure and the initial state [2]. When the outputs of all the Boolean functions at state S produce the state S itself, S is called a steady state. Since in every state every node is set to one of two Boolean values, the number of possible network states is exponential to the number of nodes. Figure 1 illustrates a simple Boolean network.

In recent years, new experimental technologies in molecular biology enabled a broader examination of gene activity in cells [3-5] and consequently, significant efforts have been invested in the application of gene regulatory networks modeling [6]. However, experimental procedures produce continuous values that do not determine conclusively the activity or inactivity of a gene. Hence, these values cannot be mapped into states of Boolean networks unambiguously, and the resulting picture of the cell state contains errors. Computational methods address this problem in various ways, for example, by using additional data such as the genomic sequences of gene promoters [7], by mapping the continuous measurements into discrete values and then optimally fitting the transformed dataset to a network model [8,9], or by using a prior distribution on states [10]. It is well recognized that an improved ability to probe the state of a cell can lead to improvement in our understanding of a broad range of biological processes.

With this motivation in mind, we propose a novel algorithm for inferring the state of a Boolean network using a continuous noisy expression dataset and the network structure, i.e., the genes and their regulators. The algorithm is based on the following idea: High expression values are more likely to correspond to a Boolean 1, while low to a Boolean 0. By combining the network structure and the expression dataset, we can estimate the likelihood of each continuous value to correspond to a Boolean value of 1 or 0. We can then update the likelihood (equivalently the expression value) of each gene accordingly and repeat the process until any further change would either (a) change a gene towards a Boolean value that is less likely for it or (b) change a gene towards a Boolean value that is as likely as the opposite Boolean value (i.e., make an arbitrary guess). The update scheme should be such that if enough updates were possible, the final probability distribution will describe the states of a Boolean network.

The next section explains how to implement this idea using the conditional entropy [11] of the network. It will be shown that changing the gene probabilities in the opposite direction of the conditional entropy gradient is equivalent to executing the inference algorithm that we outlined above. The section begins by analyzing a simple network and then extends the results to general networks.

In the ‘Testing’ section, we use simulation and real data in order to test the performance of the algorithm. We generate noisy datasets for several Boolean network structures and use a microarray time-series dataset from a study of the *Saccharomyces cerevisiae* cell cycle. We find that using the simulated datasets, the algorithm infers a large proportion of the Boolean states correctly; and using the yeast dataset, it infers Boolean states that agree with the conclusion of the study. We conclude by summarizing our results and suggesting research directions that can lead to further progress in this domain.

## Main text

### Analysis

Consider the following simple network: gene X negatively regulates gene Y. In other words, when X is active Y is inactive, and vice versa. X is also said to be a repressor of Y or to repress Y. The Boolean function that controls the value of Y is called NOT.

An experimental device can measure the states of X and Y. If a gene is active, it measures a value from a normal distribution with a large positive average *μ* and small standard deviation *σ*. If a gene is inactive, the device measures a value from a normal distribution with a negative average − *μ* and standard deviation *σ*.

The input to our problem is a series of *N* i.i.d. measurements of the genes X, Y (for example, under different stimulations given to the cells). X can be active or inactive in every measurement with equal probabilities. We are also given the structure of the network. We do not know the logic with which X regulates Y, but the values in the dataset will reflect this logic.

Our goal is to find the states of X and Y in each measurement. Clearly, we cannot always recover the ‘true’ states from every measurement, since there is a nonzero probability that the device will measure a large value for the inactive gene and, at the same time, a small value for the active gene. Nevertheless, the best strategy is to identify X as a repressor and then predict that in each pair of values the larger one corresponds to an active gene and the smaller to an inactive - the larger the difference, the higher our confidence. The inference algorithm, which we will shortly describe, will apply a generalization of this strategy. We will show that in the case of the simple network, the algorithm predicts the network states in the optimal way. Then, we will explain how it generalizes to more complex networks. Before we describe the algorithm, we need to define several random variables.

Denote the *N* measurements by C_1_, C_2_,…,C_*N*_, and the continuous values of X and Y in measurement C_*i*_ as *x*_*i*_ and *y*_*i*_, respectively. As a convention, we will use uppercase and lowercase letters to define variables that assume discrete values and continuous values, respectively. The terms measurement *i* and C_*i*_ are used interchangeably.

We define the following continuous values:

$$\begin{array}{l} \begin{array}{l} \lambda \left(x_{i}\right)=\frac{1}{1+e^{-x_{i}}}\left(\mathrm{the}\;\mathrm{logistic}\;\mathrm{function}\;\mathrm{of}\;x_{i}\right)\\ \bar{\lambda }\left(x_{i}\right)=1-\lambda \left(x_{i}\right)=1-\frac{1}{1+e^{-x_{i}}}. \end{array} \end{array}$$

The role of the logistic function is to map continuous values to probabilities. For example, if *x*_*i*_ is close to the average of its distribution *μ*, it will have a high probability to correspond to a Boolean 1, because *μ* is a large positive number. The use of the logistic function will also enable us to implement the update step in our algorithm, in which we update the probabilities of the previous iteration.

Similarly we define

$$\begin{array}{l} \lambda \left(y_{i}\right)=\frac{1}{1+e^{-y_{i}}}\\ \bar{\lambda }\left(y_{i}\right)=1-\frac{1}{1+e^{-y_{i}}}. \end{array}$$

Using these values, we define the discrete random variable [*X*;*Y*]_*i*_ ∈ {00, 10, 01, 11}:

$$\begin{array}{c} P\left({\left[X;Y\right]}_{i}=11\right)=\lambda \left(x_{i}\right)\cdot \lambda \left(y_{i}\right)\\ P\left({\left[X;Y\right]}_{i}=00\right)=\bar{\lambda }\left(x_{i}\right)\cdot \bar{\lambda }\left(y_{i}\right)\\ P\left({\left[X;Y\right]}_{i}=10\right)=\lambda \left(x_{i}\right)\cdot \bar{\lambda }\left(y_{i}\right)\\ P\left({\left[X;Y\right]}_{i}=01\right)=\bar{\lambda }\left(x_{i}\right)\cdot \lambda \left(y_{i}\right). \end{array}$$

The probability distribution of [*X*;*Y*]_*i*_ is well defined, since all probabilities are in (0,1) and sum to 1.

Since each of *x*_*i*_ and *y*_*i*_ is from one of the normal distributions *N*(*μ*,*σ*^2^), *N*(−*μ*,*σ*^2^) with a small *σ*^2^, the probabilities *P*([*X*;*Y*]_*i*_ = 11) and *P*([*X*;*Y*]_*i*_ = 00) will be small.

Similar to [*X*;*Y*]_*i*_, we can define the discrete random variable *X*_*i*_ with probability function:

$$P\left(X_{i}=1\right)=\lambda \left(x_{i}\right)\;\mathrm{and}\;P\left(X_{i}=0\right)=\bar{\lambda }\left(x_{i}\right).$$

We define the discrete random variable *Y*_*i*_ by replacing $\lambda \left(x_{i}\right),\bar{\lambda }\left(x_{i}\right)$ with $\lambda \left(y_{i}\right),\bar{\lambda }\left(y_{i}\right)$ in the definition of *X*_*i*_.

The discrete random variables that we defined so far correspond to specific experiments. We also need to define discrete random variables that correspond to the set of experiments as a whole. For example, such variables would answer the question: What is the probability of seeing *X* = 1 and *Y* = 0 in the whole dataset? In order to do that, note that as *σ*^2^ becomes smaller and the number of measurements *N* larger, by the law of large numbers:

$$\begin{array}{l} \frac{\sum_{i=1}^{N}P\left({\left[X;Y\right]}_{i}=10\right)}{N}\approx \frac{1}{2},\frac{\sum_{i=1}^{N}P\left({\left[X;Y\right]}_{i}=01\right)}{N}\approx \frac{1}{2}\\ \frac{\sum_{i=1}^{N}P\left({\left[X;Y\right]}_{i}=11\right)}{N}\approx 0,\frac{\sum_{i=1}^{N}P\left({\left[X;Y\right]}_{i}=00\right)}{N}\approx 0, \end{array}$$

which is what one expects intuitively - either *X* is active and *Y* is inactive, or vice versa, but they cannot both be active or inactive in the same measurement, because *X* represses *Y*. Although it is possible to have a high probability *P*([*X*;*Y*]_i_ = 00) for some *i*, such deviations will have little effect on the average of the *N* samples. Hence, we define a variable [*X*;*Y*] ∈ {00, 01, 10, 11} with a distribution that is an average of the probabilities of the variables [*X*;*Y*]_*i*_.

Since *X* can be inactive or active in any measurement with equal probabilities, similarly to [*X*;*Y*] we define the variable *X* using the distribution

$$\frac{\sum_{i=1}^{N}P\left(X_{i}=1\right)}{N}\approx \frac{\sum_{i=1}^{N}P\left(X_{i}=0\right)}{N}\approx \frac{1}{2}$$

and in a similar way a discrete random variable *Y*. Note that the probability of [*X*;*Y*] is an estimation of the joint probabilities of *X* and *Y*, *P*(*X*,*Y*).

How can we infer the probabilities of variables that do not conform to the *X* → *Y* network, for example, when *x*_*i*_ and *y*_*i*_ are both positive? We can use the average of all the samples, which is rather accurate, and estimate the probabilities of *X*_*i*_ = 1 and *Y*_*i*_ = 1. Then we will correct the values of *x*_*i*_ and *y*_*i*_ accordingly. This estimation and correction process is in fact equivalent to changing *x*_*i*_ and *y*_*i*_ in the opposite direction of the gradient of the conditional entropy *H*(*Y*|*X*). We have defined the probability distributions *P*(*X*), *P*(*Y*), *P*(*X*,*Y*) as functions of the continuous values *x*_*i*_, *y*_*i*_. We can therefore partially derive the conditional entropy *H*(*Y*|*X*) according to each continuous value and obtain the gradient ∇*H*(*Y*|*X*). This leads to the following algorithm:

Algorithm 1: State Inference

We now show that the algorithm obtains the desired solution for our simple network. More specifically, if *y*_*i*_ >*x*_*i*_, then *λ* (*x*_*i*_) will approach 0 and *λ*(*y*_*i*_) will approach 1 and vice versa.

First, in order to compute the gradient, we use the chain rule for conditional entropy: *H*(*Y*|*X*) = *H*(*Y*,*X*) − *H*(*X*).

It is easy to see [12] that

$$\begin{array}{ll} \frac{\partial H\left(Y,X\right)}{\partial x_{i}} & =\underset{\left(X,Y\right)\in \left\{00,01,10,11\right\}}{\sum }\frac{\partial P\left(X,Y\right)}{\partial x_{i}}\cdot \log\left(P{\left(X,Y\right)}^{-1}\right)\\ & \;-\underset{\left(X,Y\right)\in \left\{00,01,10,11\right\}}{\sum }\frac{\partial P\left(X,Y\right)}{\partial x_{i}}\\ & =\underset{\left(X,Y\right)\in \left\{00,01,10,11\right\}}{\sum }\frac{\partial P\left(X,Y\right)}{\partial x_{i}}\cdot \log\left(\frac{P{\left(X,Y\right)}^{-1}}{e}\right)\\ & \;\left({\mathrm{for}\;\log}_{e}\right). \end{array}$$

Expanding the partial derivative we have

$$\begin{array}{ll} \frac{1}{N} & \left[\begin{array}{l} \begin{array}{c} \left(\lambda \left(x_{i}\right)\cdot \left(1-\lambda \left(x_{i}\right)\right)\cdot \lambda \left(y_{i}\right)\right)\cdot \log\frac{P{\left(\left(X,Y\right)=\left(1,1\right)\right)}^{-1}}{e}+\\ \left[\lambda \left(x_{i}\right)\cdot \left(1-\lambda \left(x_{i}\right)\right)\cdot \left(1-\lambda \left(y_{i}\right)\right)\right]\cdot \log\frac{P{\left(\left(X,Y\right)=\left(1,0\right)\right)}^{-1}}{e}\\ -\left(\lambda \left(x_{i}\right)\cdot \left(1-\lambda \left(x_{i}\right)\right)\cdot \lambda \left(y_{i}\right)\right)\cdot \log\frac{P{\left(\left(X,Y\right)=\left(0,1\right)\right)}^{-1}}{e} \end{array}\\ -\left[\lambda \left(x_{i}\right)\cdot \left(1-\lambda \left(x_{i}\right)\right)\cdot \left(1-\lambda \left(y_{i}\right)\right)\right]\cdot \log\frac{P{\left(\left(X,Y\right)=\left(0,0\right)\right)}^{-1}}{e} \end{array}\right]\\ & =\frac{1}{N}\left[\left[\lambda \left(x_{i}\right)\cdot \left(1-\lambda \left(x_{i}\right)\right)\right]\cdot \left(\log\underset{X=1,Y\in \left\{0,1\right\}}{\prod }{\left(\frac{P\left(X,Y\right)}{e}\right)}^{-P\left(Y_{i}=Y\right)}-\log\underset{X=0,Y\in \left\{0,1\right\}}{\prod }{\left(\frac{P\left(X,Y\right)}{e}\right)}^{-P\left(Y_{i}=Y\right)}\right)\right]\\ & \begin{array}{ll} =\frac{1}{N}\left[\left[\lambda \left(x_{i}\right)\cdot \left(1-\lambda \left(x_{i}\right)\right)\right]\cdot \log\left(\frac{\prod_{X=0,Y\in \left\{0,1\right\}}P{\left(X,Y\right)}^{P\left(Y_{i}=Y\right)}}{\prod_{X=1,Y\in \left\{0,1\right\}}P{\left(X,Y\right)}^{P\left(Y_{i}=Y\right)}}\right)\right] & \left(*\right). \end{array} \end{array}$$

The direction of change in *x*_*i*_ (positive or negative, i.e., towards Boolean 1 or Boolean 0) will be determined by the ratio within the log. If this ratio is greater than 1, the direction of change will be negative (because the change is in the opposite direction of the gradient). If it is smaller than 1, the change will be positive.

The expression (*) expresses three properties of the data:

1. How certain we are in *x*_*i*_. If *x*_*i*_ is very high or very low, the whole expression, and the change it implies to *x*_*i*_, will be small. This is a result of the factor [*λ*(*x*_*i*_) · (1 − *λ*(*x*_*i*_))] that has its maximum at *λ*(*x*_*i*_) = 0.5 and approaches 0 when *λ*(*x*_*i*_) approaches 1 or 0.

2. The more likely Boolean value to assign to *y*_*i*._ The exponent of *P*(*X,Y*)^*P*(*Yi* = *Y*)^) will decrease the weight of the probability *P*(*X*,*Y*) in the ratio if *P*(*Y*_*i*_ = *Y*) is low, and vice versa.

3. The more likely Boolean (*X*,*Y*) vectors. For example, if *P*(*Y*_*i*_ = 0) ≈ 0, we will have within the log a ratio between *P*(*X* = 0, *Y* = 1) and *P*(*X* = 1, *Y* = 1). If *P*(*X* = 0, *Y* = 1) is more likely, the ratio will be greater than 1; and if *P*(*X* = 1, *Y* = 1) is more likely, it will be smaller than 1.

A symmetric expression can be developed for *y*_*i*_. Note that since all regulator values are equally likely, the term $\frac{\partial H\left(X\right)}{\partial x_{i}}$ is 0 (otherwise it negates the bias).

**Figure 2:**
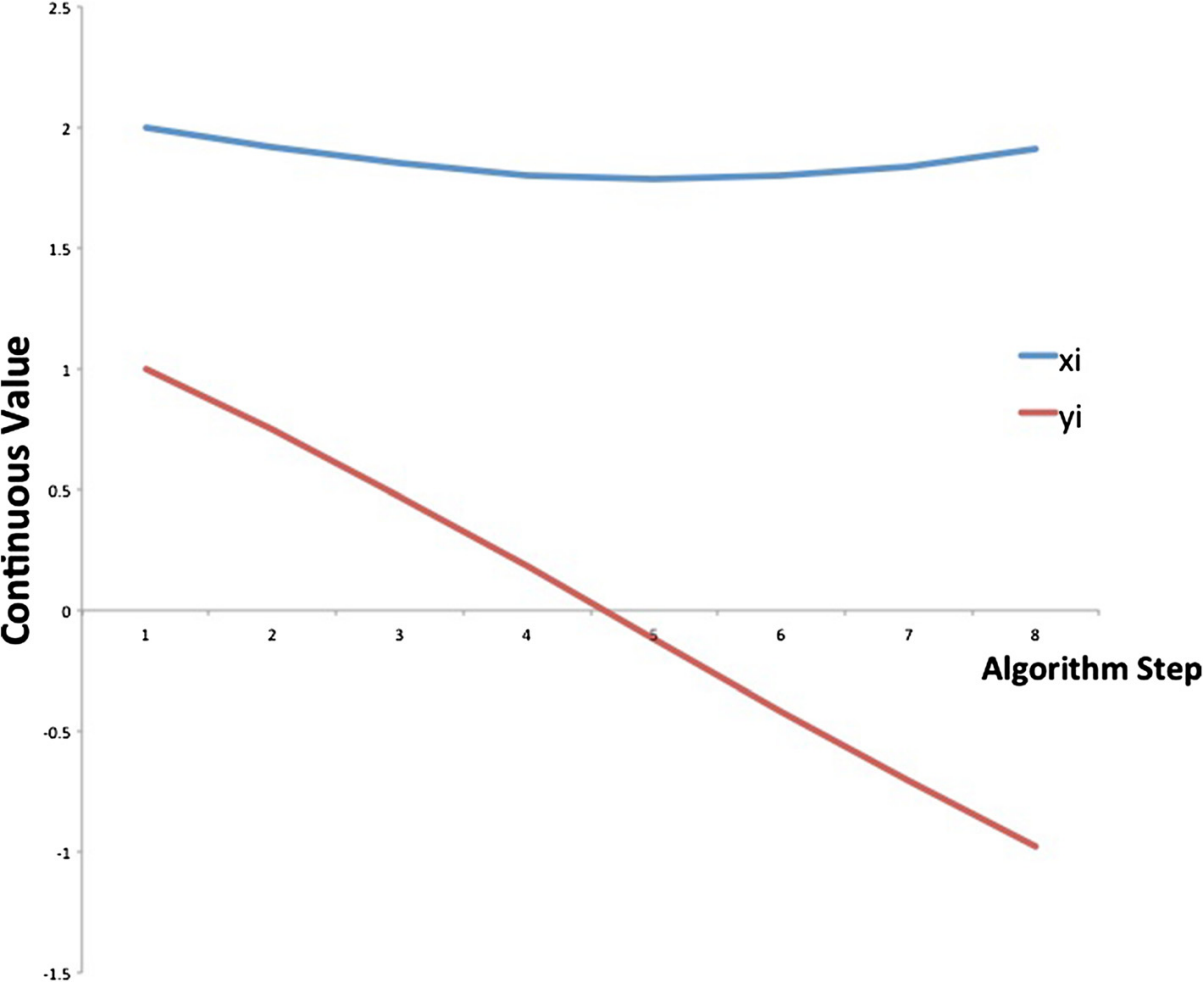
**Changes in *x***_***i***_**, *y***_***i ***_**in eight consecutive gradient descent steps.** We set *P*(*XY* = 01) = *P*(*XY* = 10) = 0.49; and *P*(*XY* = 11) = *P*(*XY* = 00) = 0.01. Since *x*_*i*_ is larger than *y*_*i*_ in the beginning, it is hardly reduced. In contrast, *y*_*i*_ is reduced sharply to a negative value. The inferred states for measurement *i* are gene X is active gene Y is inactive.

Now assume that *P*((*X*,*Y*) = (1,0)) = *P*((*X*,*Y*) = (0,1)) = 0.49; and *P*((*X*,*Y*) = (0,0)) = *P*((*X*,*Y*) = (1,1)) = 0.01. We look at measurement *i* in which *x*_*i*_ = 2 and *y*_*i*_ = 1 and plot the changes to *x*_*i*_, *y*_*i*_ in eight consecutive steps of the algorithm (Figure 2). We choose *δ* = *N* and therefore the constant 1/*N* is canceled out. As can be seen in the figure, *x*_*i*_ does not change significantly, while *y*_*i*_ is reduced sharply to a negative value. This is in agreement with our optimal solution scheme for the simple *X* → *Y* network.

We used a very simple network in order to explain the principles of our algorithm, and we now turn to more complex networks. Any network can be described by a directed graph G(V,E), where the set of nodes V contains a node for every gene, and the set of edges E contains edges from every regulator to each of its targets. The entropy of every node *Y*_*i*_ is conditional on its set of regulators **X**_*Yi*_. The conditional entropy of the network becomes $\sum_{i=1}^{\left|V\right|}H\left(Y_{i}|X_{Y_{i}}\right).$

The dataset of more complex networks may contain steady states, like in the case of the simple network, but it may also include longer trajectories. In the latter case, if two measurements *i*, *i* + 1 correspond to two consecutive states in a trajectory, the value of *y*_*i* + 1_ should be taken from C_*i* + 1_ and the values *x*_*i*_ of its regulators from C_*i*_.

In the simple network that we discussed so far, V contains two nodes, one for gene X and one for gene Y, and E contains one directed edge from the node of X to the node of Y. Each measurement is a vector of size 2, (*x*_*i*,_*y*_*i*_). For calculating (*), we needed to find the probability *P*(*X*,*Y*) of vectors of size 2.

In the general case, in order to derive the conditional entropy of the network by the value *x*_*i*_ of one of the regulators *X* at the measurement *i*, we need to find the probability of a Boolean assignment to vectors of arbitrary size. We can do this in the same way as we did for *P*([*X*;*Y*]_*i*_) - by multiplying the individual probabilities of the vector entries. The probability of seeing a Boolean vector in the dataset as a whole is again an average of its probabilities in the *N* measurements.

Denote by *M*_*x*_ the number of targets that *X* regulates. Denote by ${\vec{Z}}_{j}$, a Boolean assignment to **X**_*Yj*_ ∪ {Y_*j*_}/ *X*, where *Y*_*j*_ is the *j*th target of *X*, and **X**_*Yj*_ is the set of regulators of *Y*_*j*_, at the *i*th measurement. Denote as $\vec{Z}$ any Boolean vector of size $\left|{\vec{Z}}_{j}\right|$. We generalize the derivative by *x*_*i*_ given by (*) as follows:

$$\begin{array}{l} \;\frac{1}{N}\cdot \left[\left(\lambda \left(x_{i}\right)\cdot \left(1-\lambda \left(x_{i}\right)\right)\right)\cdot \log\left(\frac{\prod_{X=0,\vec{Z}\in {\left\{0,1\right\}}^{\left|{\vec{Z}}_{j}\right|},1\leq j\leq M_{X}}P{\left(X,\vec{Z}\right)}^{P\left({\vec{Z}}_{j}=\vec{Z}\right)}}{\prod_{X=1,\vec{Z}\in {\left\{0,1\right\}}^{\left|{\vec{Z}}_{j}\right|},1\leq j\leq M_{X}}P{\left(X,\vec{Z}\right)}^{P\left({\vec{Z}}_{j}=\vec{Z}\right)}}\right)\right].\\ \;\left(**\right) \end{array}$$

The expression (**) determines the change to *x*_*i*_ in the same way as (*), taking into account all the targets of gene X in the network. If X is itself a target of other regulators, then *M*_*x*_ increases by 1, and ${\vec{Z}}_{M_{x}+1}$ will correspond to a Boolean assignment to the regulators of X at measurement *i*.

Note that if we decrease the step size of the gradient descent *δ* by a factor C, the change in the *x*_*i*_ values $-\delta \cdot \nabla \sum_{i=1}^{\left|V\right|}H\left(Y_{i}|X_{Y_{i}}\right)$ will decrease by a factor of C. However, since the logistic function maps the *x*_*i*_ values to the finite interval (0,1), equal probabilities λ(*x*_*i*_) = *P*(*X*_*i*_ = 1) may not change by the same factor. For a ratio within the log in (**) that is very large for some *x*_*i*_, and smaller for another *x*_*j*_, the change in *P*(*X*_*j*_) as a result of decreasing *δ* can remain large while the change in *P*(*X*_*i*_) becomes small. In addition, if the change in the total entropy becomes very small as a result of decreasing *δ*, the algorithm will proceed to step 4.

It may be the case that the dataset is not sufficiently informative for inferring all the states. For example, if in the simple *X* → *Y* network *x*_*i*_ = *y*_*i*_, the algorithm will change both values to 0. On the other hand, if all *x*_*i*_ and *y*_*i*_ are different, there are always parameters *τ*, *δ* for which the algorithm will change all *x*_*i*_ and *y*_*i*_ to have opposite signs, and *H*(*Y*|*X*) will approach 0. A situation as the former can also occur in more complex networks. We would like to prove that if it does not occur, i.e., if the dataset is informative enough, our algorithm will infer the states of a Boolean network. This is shown by the following theorem:

**Theorem 1:***Let G =* (*V*,*E*) *be a graph that describes the structure of a Boolean network and D a dataset of N measurements.*

*Let X*_*Y*_*be a set of nodes that regulate some node Y, i.e., ∀ X′ ∈ X*_*Y*_*, (X′ → Y) ∈ E*

*Denote by*${\vec{X}}_{Y_{i}}$*an assignment of Boolean values to the nodes in X*_*Y*_*at measurement i. Similarly, Y*_*i*_*is a Boolean assignment to Y at measurement i.*

*If the algorithm converges to a global minimum and updates dataset D to become D′, then for any two measurements i,j in D′: *$P\left({\vec{X}}_{Y_{i}}={\vec{X}}_{Y_{j}}\wedge Y_{i}\neq Y_{j}\right)=0.$

#### Proof

The conditional entropy of the network is a sum of conditional entropies. Since conditional entropy is always nonnegative, the global minimum is reached when the conditional entropy of the network is 0, and every term in the sum is also 0.

The conditional entropy of gene Y and its set of regulators *X*_*Y*_can be written as

$$H\left(Y|X_{Y}\right)=-\underset{Y\in \left\{0,1\right\},{\vec{X}}_{Y}\in {\left\{0,1\right\}}^{\left|X_{Y}\right|}}{\sum }P\left({\vec{X}}_{Y}\right)\cdot P\left(Y|{\vec{X}}_{Y}\right)\cdot \log\left(P\left(Y|{\vec{X}}_{Y}\right)\right).$$

Since the log is non-positive and the probabilities are non-negative, *H*(*Y*|*X*_*Y*_) reaches its minimum when for every $\left(Y,{\vec{X}}_{Y}\right)$ either $P\left({\vec{X}}_{Y}\right)=0$, $P\left(Y|{\vec{X}}_{Y}\right)=0$, or $P\left(Y|{\vec{X}}_{Y}\right)=1$.

If $P\left({\vec{X}}_{Y}\right)=0$, the value ${\vec{X}}_{Y}$ of the regulators never occurs in the data.

Otherwise, if $P\left(Y=1|{\vec{X}}_{Y}\right)=0$, then since $\sum {}_{Y\in \left\{0,1\right\}}P\left(Y|{\vec{X}}_{Y}\right)=1$ it must hold that $P\left(Y=0|{\vec{X}}_{Y}\right)=1$. Similarly, if $P\left(Y=1|{\vec{X}}_{Y}\right)=1$ then $\;P\left(Y=0|{\vec{X}}_{Y}\right)=0$.

Hence, for a specific assignment ${\vec{X}}_{Y}$ of the regulators, the target *Y* is either 0 or 1 but never both.□

To summarize the analysis section, we showed that the algorithm infers the states of a simple network optimally if the dataset is informative enough. We then generalized the inference process to general networks, and showed that if the algorithm converges it will infer the states of a Boolean network.

In the ‘Testing’ section, we test the algorithm using simulation and real microarray expression data.

### Testing

#### Boolean networks with two regulators per node

**Figure 3:**
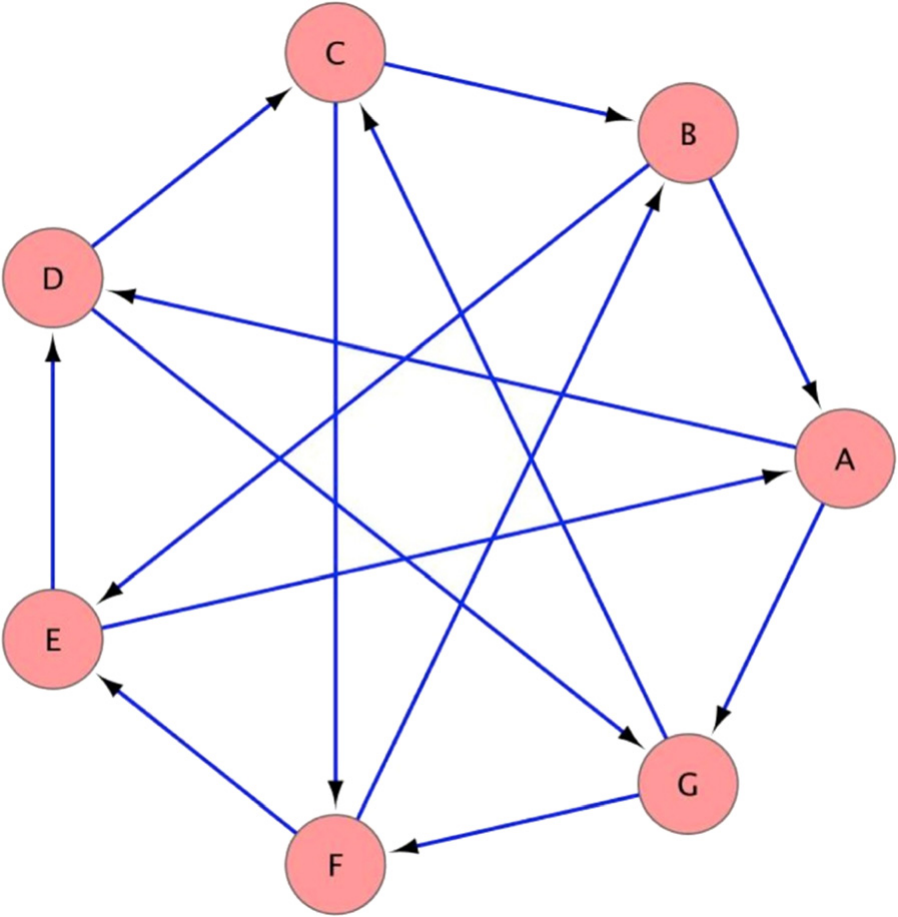
**The structure of the Boolean network used in the simulation.** For example, the regulator set of node A contains nodes B and E. The figure was generated using Cytoscape [13].

We can evaluate the accuracy of the algorithm without bias by using a known Boolean network structure. We use the Boolean network that is illustrated in Figure 3 and generate our dataset according to the following procedure:

1. Assign logic rules to all the nodes. We use the same logic function for all the nodes - XOR in the first experiment and NOR in the second experiment. XOR's output is 1 if and only if the values of the regulator nodes differ. NOR's output is 1 if and only if the values of the regulator nodes are both 0

2. Randomly choose an initial state

3. Generate a trajectory of length 400 states

4. Convert the Boolean trajectory to a continuous trajectory as follows:

(a) Replace every Boolean 1 by a value from a normal distribution with an average of 1 and a standard deviation of 1.1

(a) Replace every Boolean 0 by a value from a normal distribution with an average of −1 and a standard deviation of 1.1

**Figure 4:**
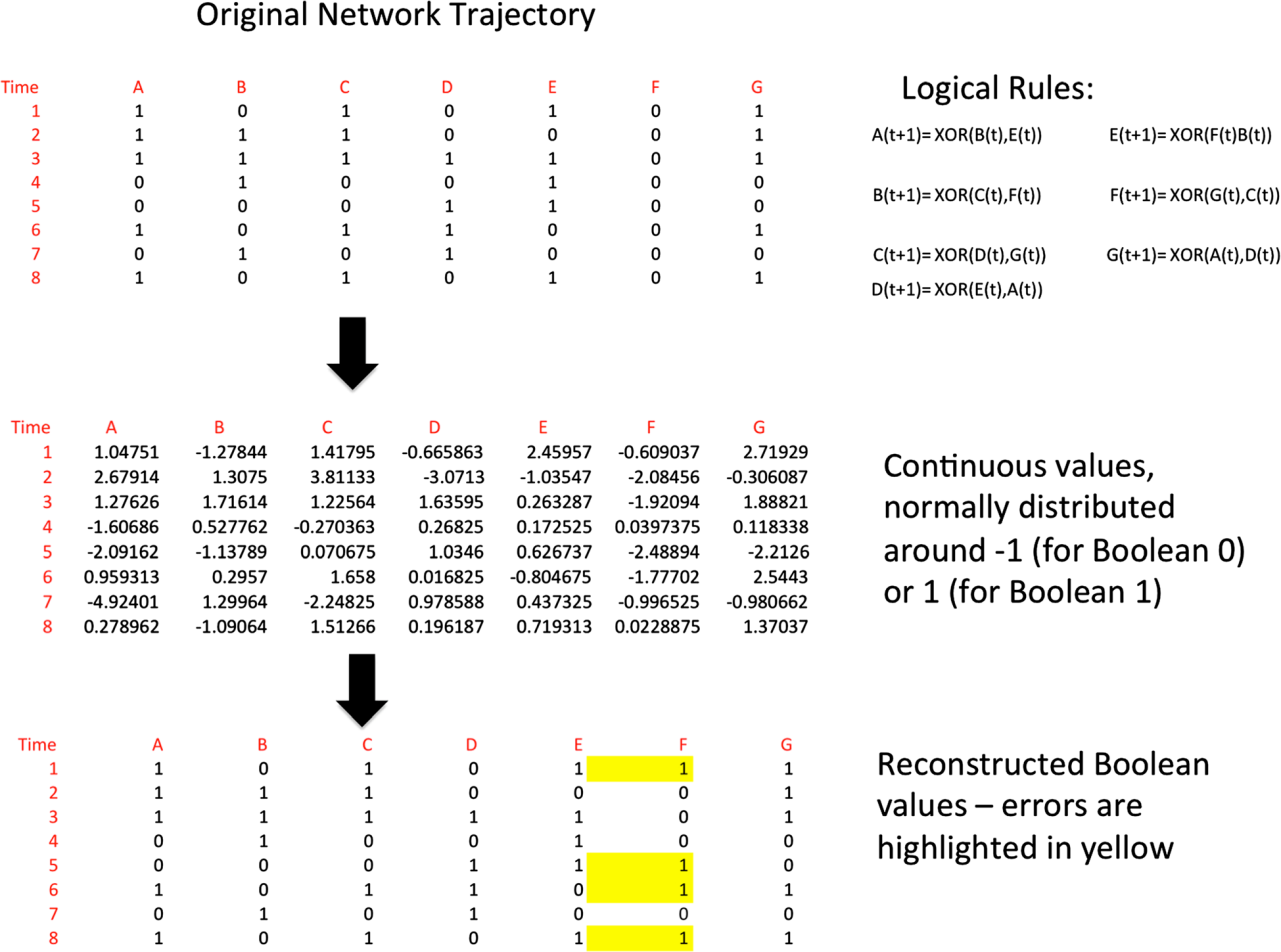
**The process by which datasets are generated in the simulation.** A Boolean trajectory is generated using a Boolean network and a set of logical rules (top). The Boolean values are translated into normally distributed continuous values (middle). The continuous values are reconstructed into Boolean values that are then compared to the original Boolean trajectory (bottom).

We use a C implementation of the algorithm as described in [12], without normalizing the continuous values. The process is illustrated in Figure 4. A trajectory of length 400 corresponds to the size of biological datasets that are available in public databases [14].

**Figure 5:**
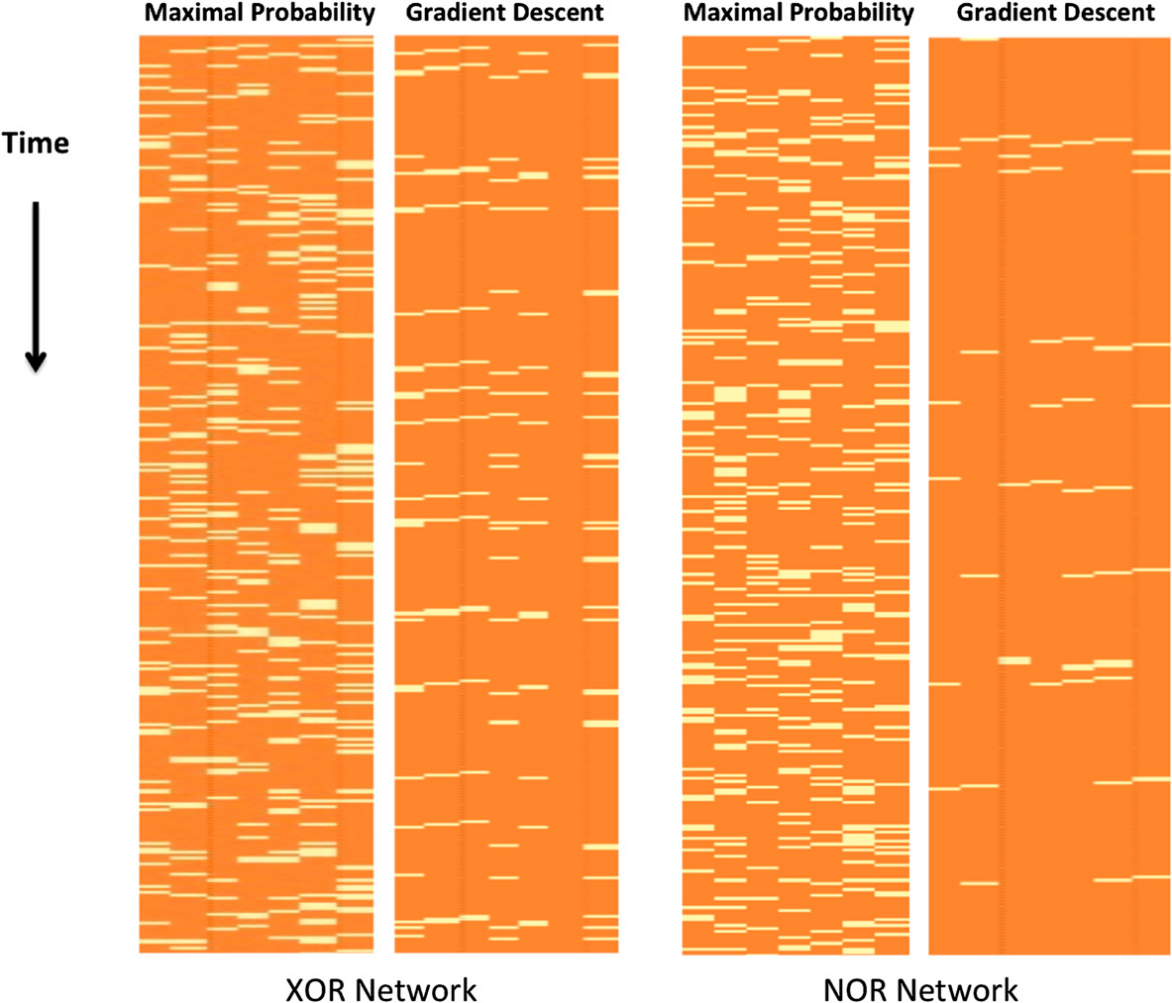
**Maximal probability reconstruction vs. gradient descent reconstruction.** Maximal probability reconstruction vs. gradient descent reconstruction of trajectories of the Boolean XOR (left) and Boolean NOR (right) networks. Rows correspond to the time points and columns to network nodes. For display purposes, only a prefix of the trajectory is shown. The yellow color represents mistakes, i.e., values which are different than the real Boolean values, and orange represents a correct value. In each of the two comparisons, the maximal probability reconstruction is presented to the left of the gradient descent reconstruction. Overall, the gradient descent is more accurate than the maximal probability reconstruction. The percentages of incorrect reconstructed values for the latter method are 17.6% (XOR) and 18% (NOR), and for the gradient descent reconstruction, 6.7% (XOR) and 2.2% (NOR).

In [15] Shmulevich and Zhang describe a mapping of continuous values to Boolean values that maps every value above some threshold to 1 and below that threshold to 0. We will compare the results of our inference process to this method, which we will refer to as ‘maximal probability reconstruction.’ The threshold that we will use is 0. Figure 5 illustrates this comparison. As can be seen in the figure, the gradient descent makes significantly less mistakes in its reconstructed trajectory. Its mistakes tend to cluster at consecutive time points, since if it makes a mistake in a regulator at time *T*, it is more likely to make mistakes in its target at time *T* + 1.

#### Boolean networks with imperfect structure

**Figure 6:**
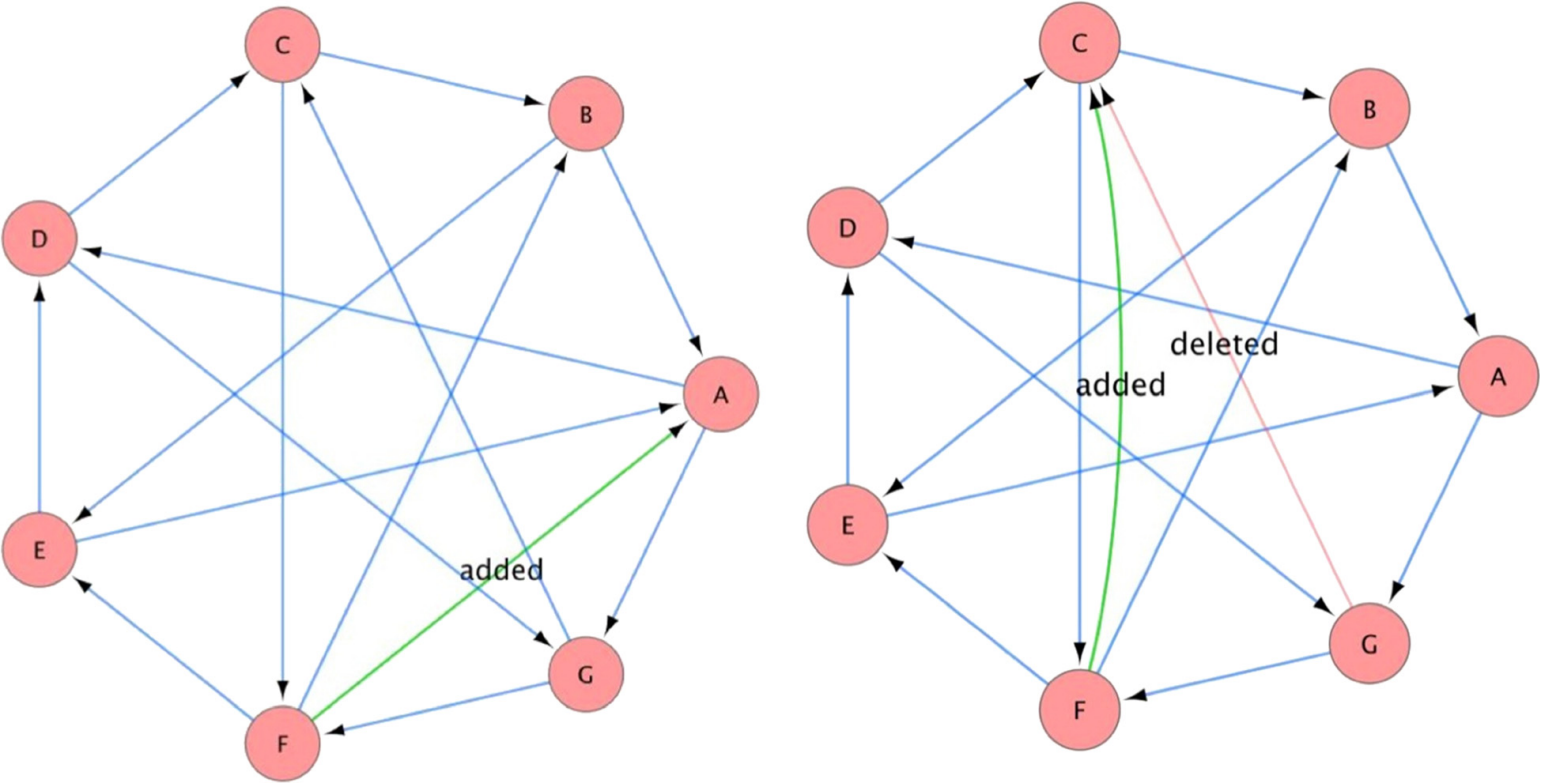
**Incorrect structures that were given to the algorithm as input.** Incorrect structures that were given to the algorithm as input with the dataset generated by the network in Figure 2. The wrong structure on the left was given as input with the dataset generated for the XOR network (see text). The wrong structure on the right was given as input with the dataset generated for the NOR network (see text). Edges that were removed (regulator sets that were changed) are colored in faded red and marked ‘deleted’. Edges that were added are colored in green and marked ‘added’. The figure was generated using Cytoscape [13].

**Figure 7:**
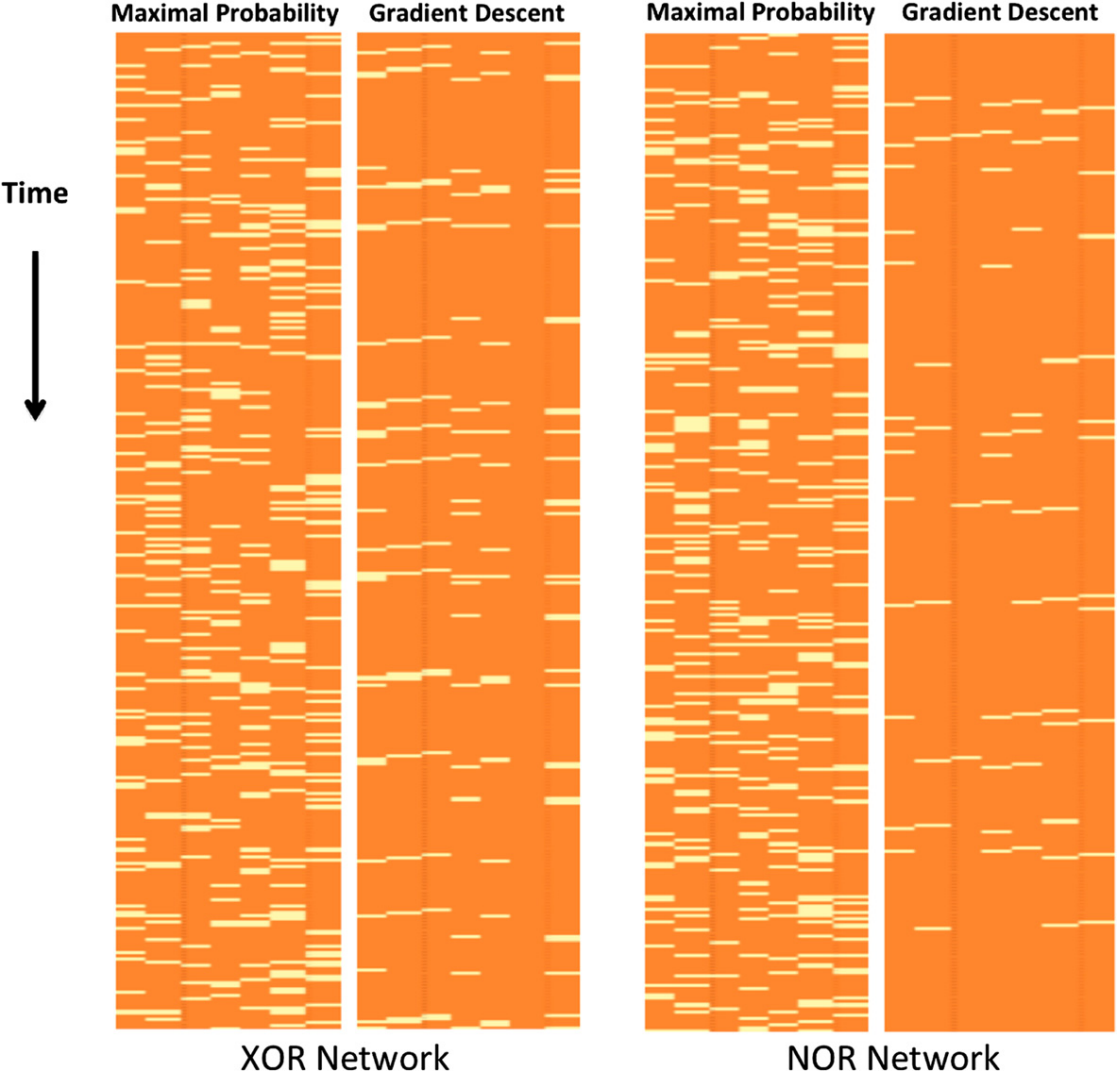
**Maximal probability reconstruction vs. gradient descent reconstruction.** Maximal probability reconstruction vs. gradient descent reconstruction of trajectories of the Boolean XOR (left) and Boolean NOR (right) networks. The gradient descent algorithm is given an inaccurate structure. The rows correspond to the time points and columns to the network nodes. For display purposes, only a prefix of the trajectory is shown. The yellow color represents mistakes, i.e., values different than the real Boolean values, and orange represents a correct value. In each of the two comparisons, the maximal probability reconstruction is presented to the left of the gradient descent reconstruction. Overall, the gradient descent is more accurate than the maximal probability reconstruction despite the imperfect structures that are given to it as input. The XOR network's trajectory reconstruction is not affected by the error in structure, while the NOR network's reconstruction is slightly less accurate. The percentages of incorrect reconstructed values for maximal probability reconstruction are 17.6% (XOR) and 18% (NOR), and for the gradient descent reconstruction, 6.7% (XOR) and 3% (NOR).

In the previous experiment, we assumed that we know the regulator set of each node. However, it is often the case that the network structure is not perfectly known, for example, some regulator set may contain incorrect nodes. Therefore, we now use the same continuous dataset, but give the algorithm an incorrect structure as input. We perform two experiments. In the first we add an incorrect node to one of the regulator sets, and in the second experiment we replace a node in a regulator set by a node that does not belong to that set. These changes are illustrated in Figure 6. As can be seen in Figure 7 when using a wrong structure, the algorithm can make more mistakes in the reconstruction of the network trajectories. However, even with an imperfect network structure, the trajectories reconstructed by the algorithm are more accurate than the maximal probability trajectories.

#### The cell cycle network of Li et al

**Figure 8:**
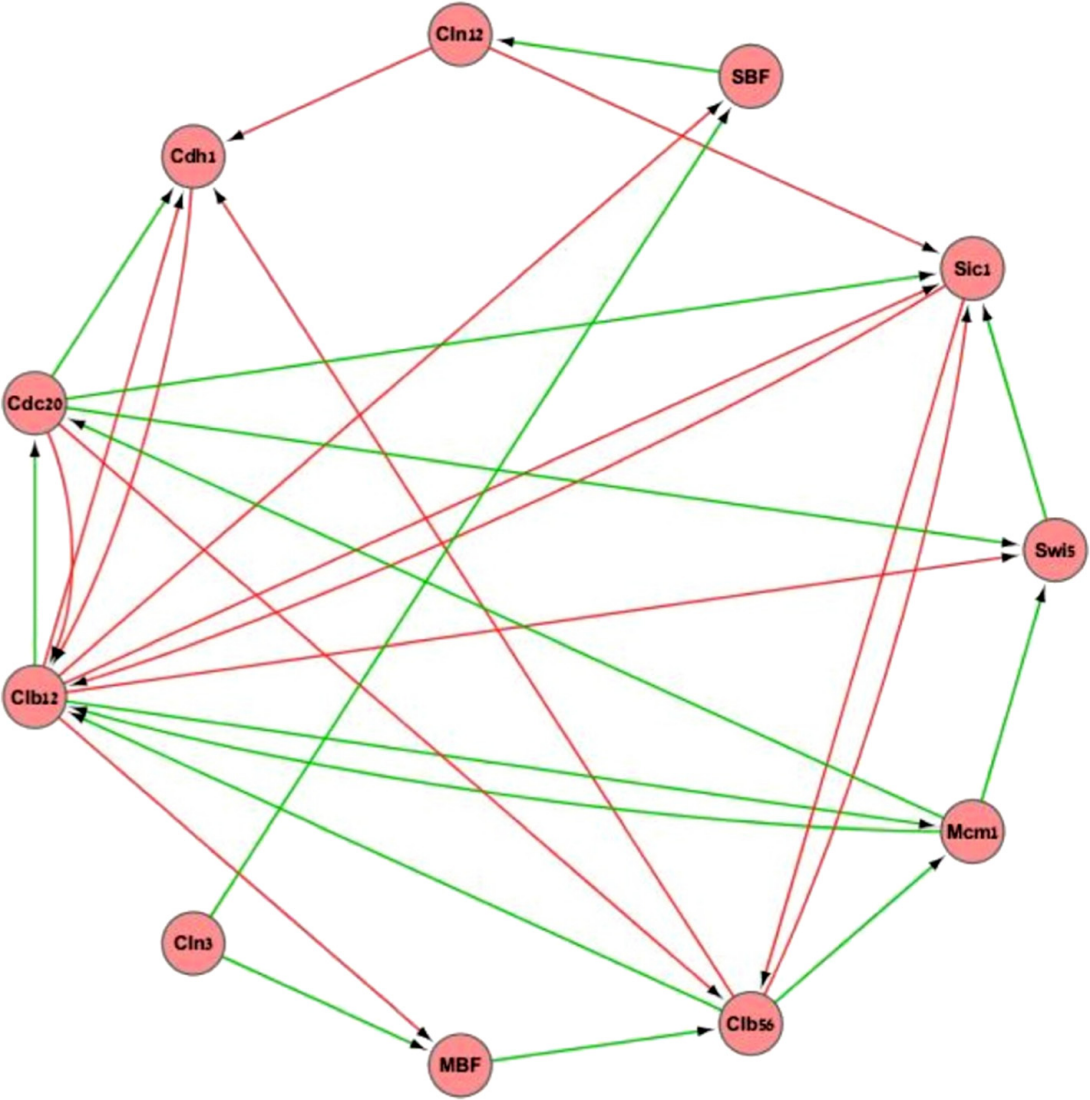
**The structure of the yeast cell cycle network of Li et al. [**[16]**].** Edges in green correspond to activators (see text), and red edges to repressors (see text). The node Cln3 has no regulators but receives an external signal that causes the network to go through the phases of the cell cycle. The figure was generated using Cytoscape [13].

The cell cycle is a process by which cells grow and multiply. It constitutes several distinct phases through which the cell grows and divides. Its daughter cells start the cycle from the first phase and so on. A gene regulatory network controls this process. Li et al. [16] created a Boolean network model of the yeast cell cycle. In their model, every node in the regulator set is assigned a repressing or an activating role and is referred to as a repressor or an activator, respectively. A node is activated by its regulator set if the sum of active activators is greater than the sum of active repressors and repressed if the former sum is lesser than the latter sum. If the sums are equal, a node either remains unchanged or is assigned a Boolean 0, meaning that without sufficient activation the gene product is degraded. Li et al. showed that the trajectories of their model converge to the first phase of the yeast cell cycle, and given an external trigger the network resumes the cycle. The network is illustrated in Figure 8.

**Figure 9:**
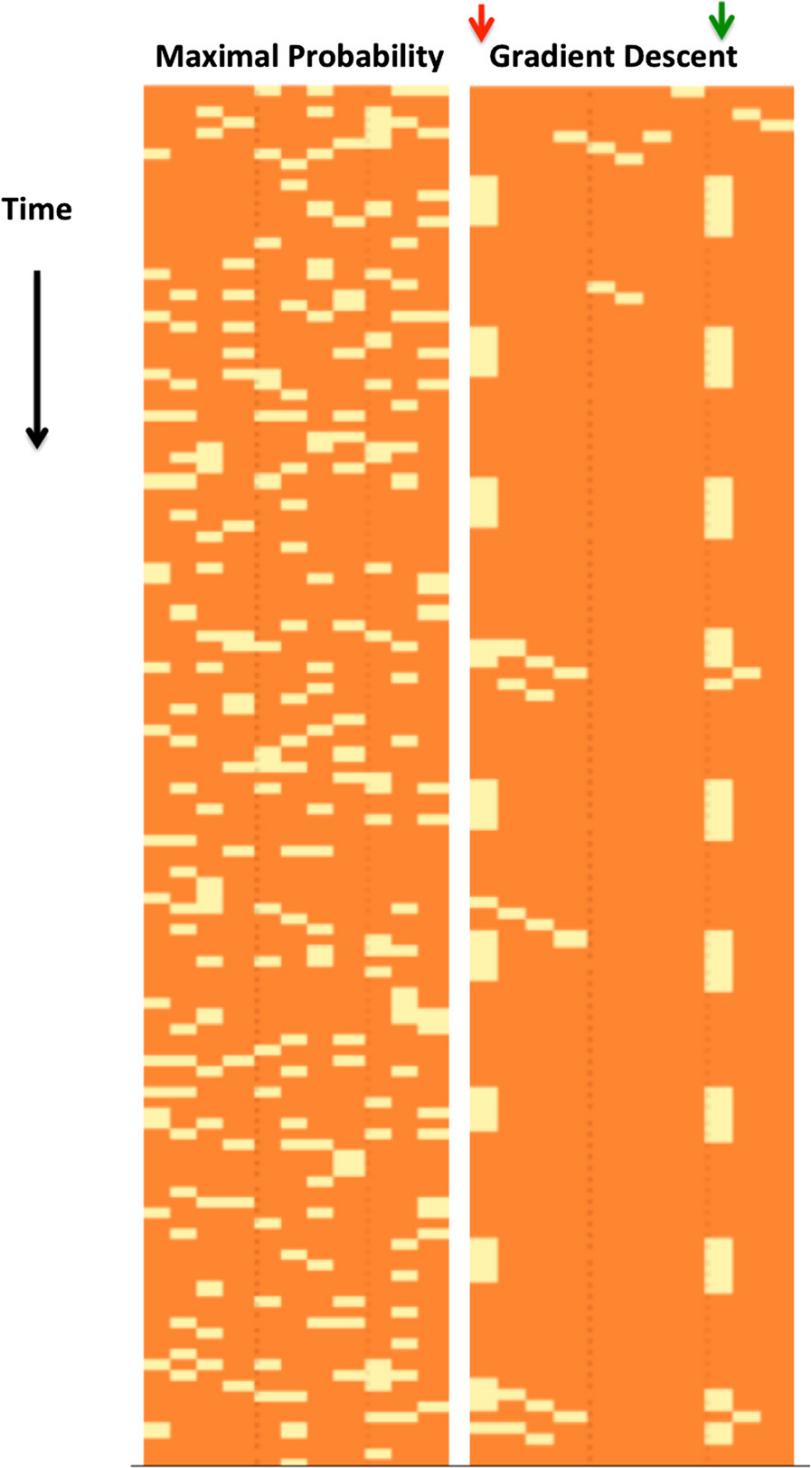
**Maximal probability reconstruction vs. gradient descent reconstruction.** Maximal probability reconstruction vs. gradient descent reconstruction of the trajectory of the yeast cell cycle network of Li et al. [16]. Rows correspond to the time points and columns to network nodes. For display purposes only a prefix of the trajectory is shown. The yellow color represents mistakes, i.e., values different than the real Boolean values, and orange represents a correct value. The maximal probability reconstruction is presented to the left of the gradient descent reconstruction. Overall, the gradient descent is more accurate than the maximal probability reconstruction. The percentages of wrongly reconstructed values for the latter method are 17.9%, and for the gradient descent reconstruction, 7.4%. The gradient descent algorithm makes more mistakes for the node Cln3 that has no regulators and receives an external input (red arrow) and for the MBF that has Cln3 in its regulator set (green arrow).

We repeated our data generation procedure for the cell cycle network of Li et al. Since this network converges to the first phase of the cell cycle and awaits a trigger to continue cycling, we provided that trigger repeatedly and generated a trajectory of length 400. The results of reconstructing the Boolean states are illustrated in Figure 9. As in the previous experiments, the reconstructed trajectory is more accurate than the maximal probability trajectory. The mistakes in this case were mainly concentrated to the node Cln3 and its direct target MBF. The reason is that when we generated the dataset, we repeatedly changed Cln3 to provide a trigger for cycling, but we did not include any regulators for Cln3 in the network structure. This creates a discrepancy between the input that we provided to the algorithm and the network behavior - the algorithm does not expect Cln3 to change its value along the trajectory if it does not have regulators.

#### Conway's game of life

Conway's game of life is composed of a square grid of cells in which each cell's Boolean value is controlled by the values of neighboring cells, and changes over time [17]. The grid can generate complex patterns that may vary significantly depending on the initial values. We modeled the game of life with grid size 7×7 as a Boolean network. Each node has 3 to 8 regulators, depending on the number of grid neighbors, and the initial state is chosen randomly. The results of reconstructing a trajectory of length 100 with the same level of noise as in previous experiments are displayed in Additional file 1: Movie 1. The left frame is the real trajectory, the middle frame is a maximal probability reconstruction, and the right frame is the gradient descent reconstruction. Boolean 1 is represented by a black cell and Boolean 0 by a white cell. As can be observed in the movie, the reconstruction algorithm makes more mistakes in the early states than in the later states. The reason for this is most likely the fact that at later states, the network enters a 3-cycle, i.e., a trajectory in which three states occur in the same order repeatedly. Since the relationships between the nodes occur more than once, the algorithm can learn these relationships and use them in reconstruction. The algorithm also identifies the existence of a 3-cycle, in the sense that it predicts a repetitive sequence of three patterns that are similar to the real patterns of the 3-cycle. In contrast, in the early time points, the states vary and do not reoccur, which makes it harder to learn some of the dependencies that play a role in generating these states. Note that most nodes have eight regulators, which means that their logic function has 256 different inputs. The number of possible network states is 2^49^.

The maximal probability reconstruction makes an error on 18.6% of the nodes. In the initial 50 states, it errs on 18.2% of the nodes, and in the last 50 states, on 19% of the nodes. The gradient descent reconstruction assigns the wrong values to 8.8% of the nodes. In the initial 50 states, its error rate is 12.8%, and in the last 50 states, 4.8%.

#### Microarray expression data

Orlando et al. [18] compared gene expression patterns in wild type yeast compared to a cyclin mutant strain. They observed that many genes are expressed in a cyclic pattern in both strains. In order to explain this observation, they suggested a Boolean network of nine transcription factors and transcription complexes. They showed that for logic functions of their choice and most initial states, the network traverses the cell cycle stages and, therefore, can explain their observation. We will use the expression data of the transcription factors and the network structure from [18] and infer the network states in wild type and mutant cells. If the states represent the cell cycle in both strains, then our analysis will support the conclusion of the study.

For the MBF, SBF, and SFF complexes, we use the expression profiles of their members STB1, SWI4, and FKH1, respectively. The dataset of [18] contains four time series of 15 microarrays for time points from 30 to 260 min, two replicates for the wild type and two for the mutant. Since all expression values are positive values, we need to map them to a symmetric range centered at 0, as the input of the simulations. However, different arrays will typically contain biases; for example, a gene can have a higher value in an array that has a higher mean expression value. Therefore, mapping two identical values from two different arrays to the same value may result in a bad estimation of the initial probabilities.

**Figure 10:**
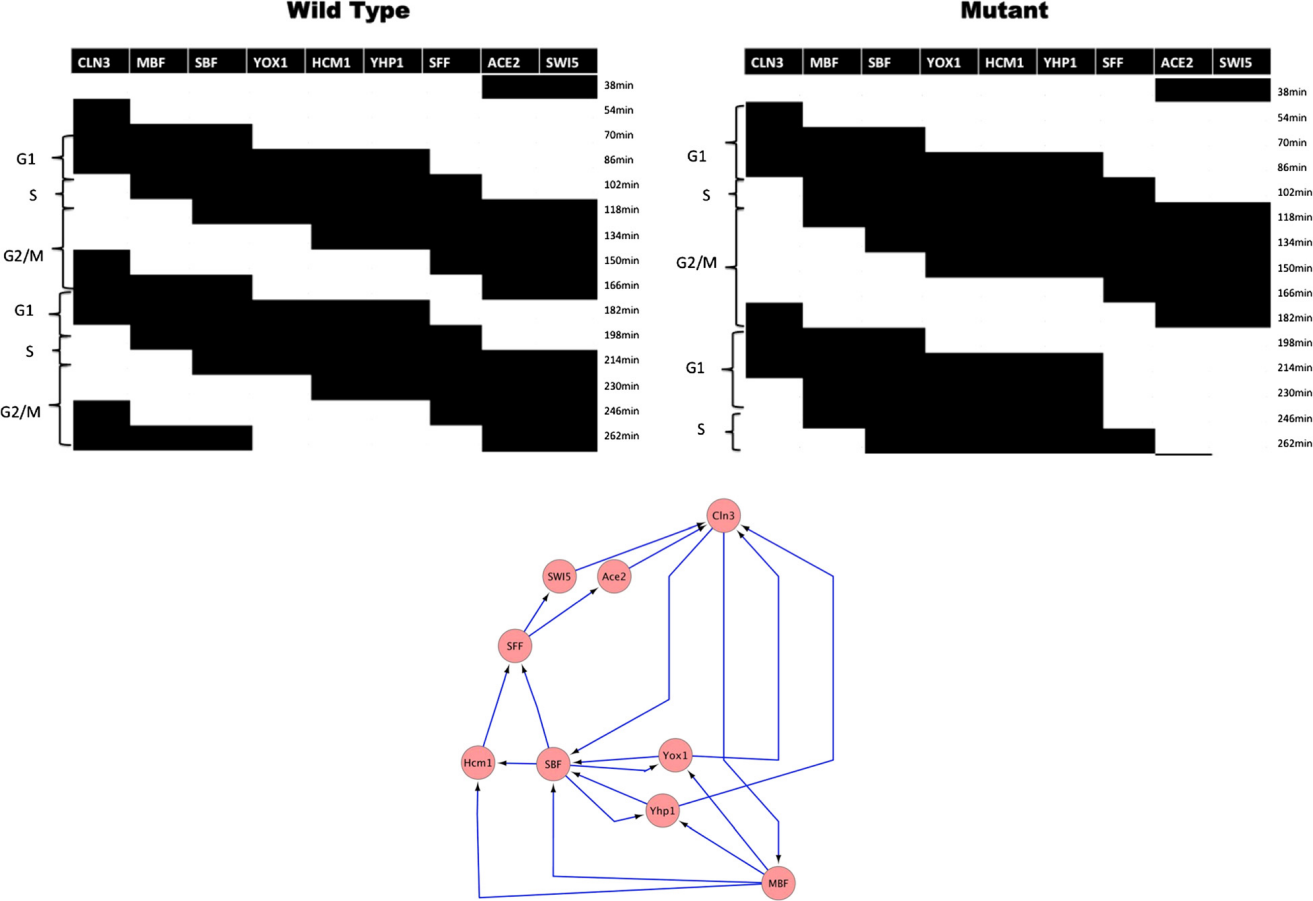
**Inferred trajectories of the Boolean network for wild type and mutant strains.** Inferred trajectories of the Boolean network from [18] for the wild type and mutant strains (one replicate is shown for each). The network structure is displayed at the bottom. Black cells correspond to a Boolean 1 and white cells to a Boolean 0. The corresponding cell cycle stages are marked at the left side of each trajectory. Both trajectories start with active ACE2 and SWI5, which are the last active factors at the completion of the previous cycle, followed activation of CLN3 and an initiation of a new cycle. The network seems to cycle a little faster in the wild type. The network structure was generated using Cytoscape [13].

Shmulevich and Zhang [15] showed that bias in different arrays can be eliminated by applying a normalization process. We use the following normalization: The network is expected to perform about two cell cycles during the measured time points. The expression levels of a gene at the 2 cycles should correlate. Based on this observation, we normalize in every replicate the genes on the first set of seven arrays and the second set of eight arrays to average 0 and unit standard deviation. Using the resulting initial probability estimates and the network structure, we apply our inference process and compare the resulting set of Boolean states with the pattern hypothesized in the study (Figure 10). As can be seen in the figure, the network performs a cyclic trajectory in both strains, while the trajectory of the mutant corresponds to a slower cell cycle. This finding is in agreement with a slower cell cycle for the mutant as reported in [18]. It also indicates that the network structure may not account for all the regulatory interactions in the network, since both networks start from the same initial state.

## Conclusions

In this study we presented a problem that arises in molecular biology, namely, that of inferring the activity of cellular network components given noisy measurements, and defined it as mapping continuous measurements to Boolean network states. We developed an algorithm that given a network structure infers its Boolean states from a dataset of continuous measurements. Our results show that the algorithm can successfully reconstruct Boolean states from inaccurate continuous data. The algorithm performs reasonably well even if the relations between the nodes of the network contain errors. We also showed that it can be used to interpret real microarray data and examine experimental hypotheses.

Our approach is highly dependent on a network structure, and when that is not available, methods that rely solely on expression should be used [15,19]. We did not define a concept of prior knowledge, which has been used in various works to integrate information into Bayesian models [20,21]. While this makes our method arguably less flexible, it also exempts us from the need to define prior distributions. Finally, the algorithm is defined for deterministic Boolean networks, in contrast to probabilistic Boolean networks that may better express biological noise [22].

Further research could improve inference accuracy and explore various aspects of the problem. One such aspect is the amount of information about the network trajectory that is lost due to noise. In the simple network that we described in the analysis section, the proportion of information that will be lost is the sum of probabilities of two events:

$$\begin{array}{l} P\left(x_{i}\sim N\left(\mu ,\sigma^{2}\right)\wedge y_{i}\sim N\left(-\mu ,\sigma^{2}\right)\wedge x_{i}\leq y_{i}\right)\\ \;+P\left(x_{i}\sim N\left(-\mu ,\sigma^{2}\right)\wedge y_{i}\sim N\left(\mu ,\sigma^{2}\right)\wedge x_{i}\geq y_{i}\right). \end{array}$$

When one of these two events occurs, it is impossible to reconstruct the original states of *X* and *Y*. In more complex networks, information loss is a more complex. Determining an upper limit on the number of Boolean values that can be recovered given a certain amount of noise may prove insightful.

Another aspect that should be investigated is how to choose parameters that optimize the performance of the algorithm, such as the parameters of the logistic function or the step size *δ* and threshold *τ* of the gradient descent.

As Boolean networks can generate a diverse range of dynamic behaviors, the accuracy of reconstructing trajectories that arise in different dynamic regimes should also be characterized. For example, are chaotic trajectories harder to reconstruct then those that display order? More simulation tests can better define the relationships between the quality of data and different classes of networks.

Current experimental techniques produce an ever-greater number of measurements, and there is a pressing need for methods that will enable researchers to interpret it accurately and without bias. An accurate method for inferring the state of a cell can translate this richness of data into important discoveries.

## Competing interests

The author declares that he has no competing interests.

## Supplementary Material

Additional file 1 **Movie 1.** Reconstruction of a trajectory of Conway’s Game of Life. The left frame is the real trajectory, the middle frame is a maximal probability reconstruction, and the right frame is the gradient descent reconstruction. Boolean 1 is represented by a black cell and Boolean 0 by a white cell. Click here for file
